# Complete Genome Sequence of the Virulent Aeromonas salmonicida subsp. masoucida Strain RFAS1

**DOI:** 10.1128/genomeA.00470-18

**Published:** 2018-05-31

**Authors:** Ahran Kim, Thanh Luan Nguyen, Do-Hyung Kim

**Affiliations:** a Department of Aquatic Life Medicine, Pukyong National University, Busan, Republic of Korea

## Abstract

Here, we report the complete genome sequence of the pathogenic Aeromonas salmonicida subsp. masoucida strain RFAS1, isolated from black rockfish and showing signs of furunculosis. Sequencing with the PacBio platform yielded a circular chromosome of 4,783,004 bp and two plasmids (70,968 bp and 63,563 bp) harboring 4,411, 67, and 71 protein-coding genes, respectively.

## GENOME ANNOUNCEMENT

Aeromonas salmonicida is a Gram-negative bacterial pathogen that causes furunculosis in a diverse range of freshwater and marine fish worldwide (1, 2). Aeromonas salmonicida subsp. masoucida strain RFAS1 was isolated from the kidney and spleen of black rockfish (Sebastes schlegeli) showing typical external signs of furunculosis, such as ulcer lesions and hemorrhages over the operculum. Han et al. (3) also showed that strain RFAS1 was beta-hemolytic against erythrocytes of black rockfish. It was able to multiply in the serum, causing fish death in an artificial challenge test. Antimicrobial susceptibility tests according to protocol of the Clinical and Laboratory Standards Institute (4) have shown that this strain is resistant to multiple antibiotics, including amoxicillin, ampicillin, cephalexin, and sulfamethoxazole.

Genomic DNA of RFAS1 was extracted using the DNeasy blood and tissue kit (Qiagen, Streetville, ON, Canada) according to the manufacturer’s instructions. Whole-genome sequencing was conducted using the PacBio RS II P6-C4 chemistry platform (Pacific Biosciences, Menlo Park, CA, USA), which is a single-molecule real-time (SMRT) cell sequencing system. Raw sequences generated by the PacBio RS II P6-C4 chemistry platform were analyzed with SMRT Analysis version 2.3.0, and the Hierarchical Genome Assembly Process 2 (HGAP2) was used for assembly and polishing, which resulted in three contigs with a total size of 4,917,535 bp and a G+C content of 58.27%. A total of 4,579 genes, including 120 tRNAs, 31 rRNAs, and 4 noncoding RNAs, in the RFAS1 genome were predicted and annotated with the NCBI Prokaryotic Genome Annotation Pipeline (5). In addition, antibiotic resistance genes, genomic islands, and virulence-related genes were investigated using the Resistance Gene Identifier (RGI) in the Comprehensive Antibiotic Resistance Database (CARD) (6), IslandViewer3 (7), and the Virulence Factors Database (VFDB) (8), respectively.

This genome contains a single circular chromosome (4,783,004 bp) with 4,411 protein-coding sequences (CDSs), as well as two plasmids, plasmid 1 (70,968 bp) with 67 CDSs and plasmid 2 (63,563 bp) with 71 CDSs. Average nucleotide identity analysis (9) showed that strain RFAS1 shared 99.97 to 100% and 99.56 to 99.79% identities with strains of A. salmonicida subsp. masoucida and A. salmonicida subsp. salmonicida, respectively. Pan-genome analysis (10) revealed 185 (16.1%) singleton genes in strain RFAS1, mainly consisting of hypothetical proteins and part of a type IV section system (T4SS). As found in the typical strain A. salmonicida A449 (11), analysis using the VFDB revealed that RFAS1 possessed various virulence-related genes such as RTX toxin (12), aerolysin (13), hemolysin (14), adhesins (A-layer [15, 16], lateral and polar flagella [11]), three type IV pilus systems (one type IV Aeromonas pilus [Tap] system, one mannose-sensitive hemagglutinin [MSHA] type system, and one fimbrial low-molecular-weight protein [Flp] pilus [17]), and four types of secretion systems, including T2SS and T6SS in the chromosome, T3SS in plasmid 2 (except toxic proteins such as AopH and AexT located in the chromosome), and T4SS in plasmid 1. Carbapenem-hydrolyzing metallo-beta-lactamase (*cph*A5) (18) related to beta-lactam resistance genes was identified in the chromosome of strain RFAS1 by the Resistance Gene Identifier (RGI) in the Comprehensive Antibiotic Resistance Database (CARD). This is the first genome reported for A. salmonicida isolated from black rockfish. It can be useful for comparative studies in the future to understand its pathogenicity and host specificity.

### Accession number(s).

The genome sequence reported here has been deposited at GenBank under the accession numbers CP017143 (chromosome), CP017144 (plasmid 1), and CP017145 (plasmid 2).
